# Polynomial algorithms for the Maximal Pairing Problem: efficient phylogenetic targeting on arbitrary trees

**DOI:** 10.1186/1748-7188-5-25

**Published:** 2010-06-02

**Authors:** Christian Arnold, Peter F Stadler

**Affiliations:** 1 Bioinformatics Group, Department of Computer Science, and Interdisciplinary Center for Bioinformatics, University of Leipzig, Härtelstraße 16-18, D-04107 Leipzig, Germany; 2 Harvard University, Department of Human Evolutionary Biology, Peabody Museum, 11 Divinity Avenue, Cambridge MA 02138, USA; 3 Max Planck Institute for Mathematics in the Sciences, Inselstraße 22, D-04103 Leipzig, Germany; 4 Fraunhofer Institute for Cell Therapy and Immunology, Perlickstraße 1, D-04103 Leipzig, Germany; 5 Santa Fe Institute, 1399 Hyde Park Rd., Santa Fe, NM 87501, USA; 6 Institute for Theoretical Chemistry, University of Vienna, Währingerstraße 17, A-1090 Wien, Austria

## Abstract

**Background:**

The *Maximal Pairing Problem *(MPP) is the prototype of a class of combinatorial optimization problems that are of considerable interest in bioinformatics: Given an arbitrary phylogenetic tree *T *and weights *ω*_*xy *_for the paths between any two pairs of leaves (*x*, *y*), what is the collection of edge-disjoint paths between pairs of leaves that maximizes the total weight? Special cases of the MPP for binary trees and equal weights have been described previously; algorithms to solve the general MPP are still missing, however.

**Results:**

We describe a relatively simple dynamic programming algorithm for the special case of binary trees. We then show that the general case of multifurcating trees can be treated by interleaving solutions to certain auxiliary *Maximum Weighted Matching *problems with an extension of this dynamic programming approach, resulting in an overall polynomial-time solution of complexity (*n*^4 ^log *n*) w.r.t. the number *n *of leaves. The source code of a C implementation can be obtained under the GNU Public License from http://www.bioinf.uni-leipzig.de/Software/Targeting. For binary trees, we furthermore discuss several constrained variants of the MPP as well as a partition function approach to the probabilistic version of the MPP.

**Conclusions:**

The algorithms introduced here make it possible to solve the MPP also for large trees with high-degree vertices. This has practical relevance in the field of comparative phylogenetics and, for example, in the context of phylogenetic targeting, i.e., data collection with resource limitations.

## Background

Comparisons among species are fundamental to elucidate evolutionary history. In evolutionary biology, for example, they can be used to detect character associations [1-3]. In this context, it is important to use statistically independent comparisons, i.e., any two comparisons must have disjoint evolutionary histories *(phylogenetic independence*). The *Maximal Pairing Problem *(MPP) is the prototype of a class of combinatorial optimization problems that models this situation: Given an arbitrary phylogenetic tree *T *and weights *ω*_*xy *_for the paths between any two pairs of leaves (*x*, *y*) (representing a particular comparison), what is the collection of pairs of leaves with maximum total weight so that the connecting paths do not intersect in edges?

Algorithms for special cases of the MPP that are restricted to binary trees and equal weights (which thus simply maximizes the number of pairs) have been described, but not implemented [2]. Since different pairs of taxa may contribute different amounts of information depending on various factors (e.g., their phylogenetic distance or the difference of particular character states), the weighted version is of considerable practical interest. A particular question of this type is addressed by *phylogenetic targeting*, where one seeks to optimize the choice of species for which (usually expensive and time-consuming) data should be collected [4]. Phylogenetic targeting boils down to two separate tasks: (1) estimation of the weight *ω*_*xy *_that measures the benefit or our amount of information contributed by including the comparison of species *x *with species *y *and (2) the identification of an optimal collection of pairs of species such that they represent independent measurements, i.e., the solution of the corresponding MPP. To date, the only publicly available software package for phylogenetic targeting [5] can handle multifurcating trees; however, the implementation uses a brute force enumeration of subsets of children and hence scales exponentially in the maximal degree.

As a consequence of the ever-increasing amount of available sequence data, phylogenetic trees of interest continue to increase in size, and large trees with hundreds or even thousands of vertices are not an exception any more [6-9]. Most large phylogenies contain a substantial number of multifurcations that represent uncertainties in the actual phylogenetic relationships. It appears worthwhile, therefore, to extend previous approaches to efficiently solve the MPP for multifurcating trees and arbitrary weights.

## Algorithms

### Definitions and Preliminaries

Let *T*(*V, E*) be a rooted (unordered) tree with a vertex set *V *= *L *∪ *J *(where *L *are the leaves of *T*, *J *its interior vertices, |*L*| the number of leaves, and |*J*| the number of interior vertices) and an edge set *E *= *V *× *V*.

Every vertex *x*, with the exception of the root r, has a unique *father*, fa(*x*), which is the neighbor of *x *closest to the root. We set fa(*r*) = ∅. Note that, given an unrooted tree without vertices with no father, we can obtain a rooted tree by subdividing an arbitrary edge with *r*. Furthermore, for each *u *∈ *J*, let chd(*u*) be the set of children of *v *(i.e., its descendants). Obviously, *y *∈chd(*u*) if and only if fa(*y*) = *u *and chd(*u*) = ∅ if and only if *v *∈ *L*. We write *T*[*v*] for the subtree rooted at *v*. Furthermore, we assume that |chd(*u*)| ≠ 1 throughout this contribution. A tree is binary if |chd(*u*)| = 2 for all *v *∈ *J*, and multifurcating if |chd(*u*)| > 2 holds for some interior vertices. Finally, let *T*[*v*, *C*] be the subtree of *T *rooted at an interior vertex *v *∈ *J*, but with only a subset *C *of its children. All subtrees *T*[*v*] with *v *∈ *chd(v*)\*C *are thus excluded from *T*[*v*, *C*].

For the purpose of this contribution, we interpret a *path *π in *T *as a sequence {*e*_1_,...,*e*_*l*_} of edges *e*_*i *_∈ *E *such that *e*_*i *_= *e*_*j *_implies *i *= *j *and *e*_*i *_∩ *e*_*i*+1 _= {*x*_*i*_} are single vertices for all 1 ≤ *i* <*l*. The vertices *x*_*0 *_∈*e*_*1 *_and *x*_*l *_∈*e*_*l *_are the endpoints of π. For two vertices *x*, *y *∈ *V*, we denote the unique path with endpoints *x *and *y *by π_*xy*_. In the following, we will frequently be concerned with paths connecting an interior vertex *u *∈ *J *with a leaf *x *∈ *L*. This path contains exactly one child of *u*, which we denote by *u*_*x*_(*u*, *x*). In the following, the array *n*(*u*, *x*) will be used to allow efficient navigation in *T*.

A *path-system *ϒ on *T *is a set of paths π such that

1. If π = π_*xy *_∈ ϒ, then *x*, *y *∈ *L *and *x *≠ *y*, i.e., every path connects two distinct leaves.

2. If π' ≠ π'', then π' ∩ π'' = ∅, i.e., any two paths in ϒ are edge-disjoint.

**Figure 1:**
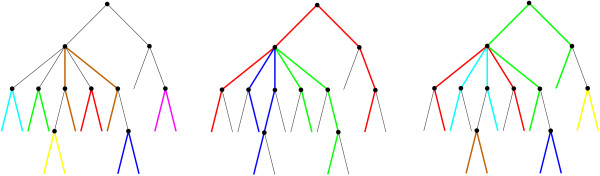
**Three different path-systems on a tree with 15 leaves**. Each path is shown in a distinctive color, and unused edges of the tree are shown as thin black lines. Clearly, no two paths share an edge, i.e., the corresponding collection of pairs of leaves is phylogenetically independent. Note that the paths are not necessarily vertex-disjoint.

Note that two paths in ϒ have at most one vertex in common (otherwise they would also share the sub-path, and therefore edges, between two common vertices). In binary trees, two edge-disjoint paths are also vertex-disjoint, since two edge-disjoint paths can only run through an interior vertex *u *with |*chd*(*u*)| ≥ 3 (see Fig. 1). Two edge-disjoint paths can share a vertex *u *in two distinct situations: (1) if both paths have *u *as the last common ancestor of their respective leaves, *u *must have at least four children, (2) if *u *is the last common ancestor for one path, while the other path also includes an ancestor of *u*, three children of *u *are sufficient. These two situations will also lead to distinct cases in the algorithms that are presented next.

Furthermore, let *ω*_*xy *_: *L *× *L *→ ℝ be an arbitrary weight function on pairs of leaves of *T*. We define the weight of a path-system ϒ as(1)

A path-system ϒ that maximizes *ω*(ϒ), i.e., a solution of the MPP, will in the following be called *optimal path-system*. It conceptually corresponds to Maddison's "maximal pairing" [2], although we describe here a more general problem (see Background and Variants). In the following sections, our main objective is to compute optimal path-systems.

### The Maximal Pairing Problem for binary trees

#### Forward recursion

In this section we reconsider the approach of [4] for the special case of binary trees. This subsumes also Maddison's [2] discussion of the special unweighted case (see section Variants). We develop the dynamic programming solution for this class of MPP using a presentation that readily leads itself to the desired generalization to multifurcating trees.

For a given interior vertex *u *∈ *J *we use the abbreviation *C*_*x *_= *C*_*x*_(*u*) = chd(*u*)\*u*_*x *_for the set of children of *u *that are not contained in the path that connects *u *with the leaf *x*. Since *T *is binary by assumption in this subsection, *C*_*x *_contains a unique vertex .

We will need two arrays (*S*, *R*) to store optimal solutions of partial problems. For each *u *∈ *V*, let *S*_*u *_be the score of an optimal path-system on the subtree *T*[*u*]. For each *u *∈ *V *and leaf *x *∈ *T*[*u*], we furthermore define *R*_*ux *_as the score of an optimal path-system on *T *[*u*] that is edge-disjoint with the path *π*_*ux*_. *R*_*ux *_can be decomposed as follows:(2)

For completeness, we set *S*_*x *_= *R*_*xx *_= 0 for all leaves *x *∈ *L*.

An optimal path-system on *T *[*u*] either consists of optimal path-systems on each of the two trees *T *[*v*] and *T*[*w*] rooted at the two children *v*, *w *∈ chd(*u*), or it contains a path π_*xy *_with endpoints *x *∈ *T*[v] and *y *∈*T*[*w*]. Thus, *S*_*u *_can be calculated as follows:(3)

Recursion (3) can then be evaluated from the leaves towards the root.

In order to facilitate the backtracing part of the algorithm, it is convenient to introduce an auxiliary variable *F*_*u*_. If an optimal score in eq.(3) is obtained by the second alternative, the pair (*x*, *y*) that led to the highest score is recorded in *F*_*u*_; otherwise, we set *F*_*u *_= ∅.

#### Backtracing

A computed optimal path-system ϒ_max _on *T *= *T *[*r*] from the forward recursions can be reconstructed by backtracing. For binary trees, this is straightforward. We start at the root *r*. In the general set, at an interior vertex *u *with *v*, *w *∈ chd(*u*), we first check whether *F*_*u *_= ∅. If this is the case, all paths π_*xy*_∈ ϒ_max _are contained within the subtrees *T*[*v*] and *T*[*w*], and we continue to backtrace in both *T*[*v*] and *T*[*w*]. If *F*_*u *_= (*x*, *y*), then π_*xy *_is added to ϒ_max_, and we need to backtrace an optimal path-system for each of the subtrees "hanging off" π_*xy*_. In other words, we need optimal path-systems for the subtrees rooted at the vertices  and  for *u *∈ π_*xy*_. These can be obtained recursively by following the decompositions of *R*_*vx *_and *R*_*wy*_, respectively, given in eq.(2).

#### Time and Space complexity

All entries *S*_*u *_for interior vertices *u *can be computed in (*n*^3^) time, because a total of n(*n *- 1) ∈ (*n*^2^) pairs of leaves have to be considered in eq.(3) and computation of each *S*_*u *_entry takes at most (*n*) time. Since we need to store the quadratic arrays *R*_*ux *_and *n*(*u*, *x*) as well as the linear arrays *S*_*u *_and *F*_u_, we need (*n*^2^) memory.

### The Maximal Pairing Problem for multifurcating trees

#### Forward recursion

In trees with multifurcations, for a path-system ϒ, more than one path can run through each vertex *m *∈ *J *with |chd(*m*)| > 2 without violating phylogenetic independence. In addition to an optimal score *S*_*u *_, we also define an optimal score *Q*_*ux *_of all path-systems  on *T*[*u*]\*T*[*u*_*x*_], i.e., of all path-systems that avoid not only the path π_*ux *_but the entire subtree *T*[*u*_*x*_], where *u*_*x *_is as usual the child of *u *along π_*ux*_. We therefore have(4)

The computation of *S*_*u *_and *Q*_*ux *_are analogous problems. In general, consider an (interior) vertex *u *∈ *J *and a subset *C *⊆ chd(*u*) of children of *u*. Our task is to compute an optimal path-system on the subtree *T*[*u*, *C*] of *T*. We first observe that any path-system on *T*[*u*, *C*] contains 0 ≤ *k *≤ ⌊|*C*|/2⌋ paths π_*k *_through *u*. Each of these paths runs through exactly two distinct children  and  of *u*. For fixed  and , the path ends in leaves  and  (Fig. 1). The best possible score contribution for the path π_*x'x'' *_is(5)

and the best possible score for a particular pair of children *v'*, *v'' *∈ *C *is therefore(6)

For the purpose of backtracing, it will be convenient to record the path *π*_*xy*_, or rather its pair of end points (*x*, *y*), that maximized  in eq.(6) in an auxiliary variable *F*_*v',v''*_.

Since there are *k *paths through *u *covering *2k *of the |*C*| subtrees, there are |*C*| - 2*k *children *v*_*l *_of *u*, with 1 ≤ *l *≤ |*C*| - 2*k*, each of which contributes to an optimal path-system with a sub-path-system that is contained entirely within the subtree *T*[*v*_*l*_]. Since these contributions are independent of each other, they are obtained by solving the MPP on *T*[*v*_*l*_], i.e., their contribution to the total score of an optimum path-system is *S*_*vl*_.

**Figure 2:**
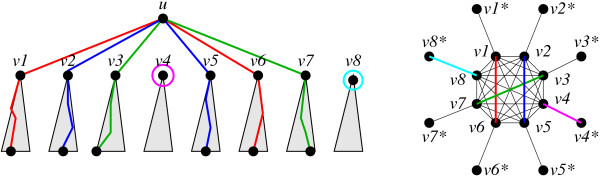
**Translation of a path-system on *T*[*u*] into a matching on the auxiliary graph Γ(chd(*u*))**.

For each subtree *T*[*u*, *C*] we therefore face the problem of determining the optimal combination of pairs and isolated children. This task can be reformulated as a weighted matching problem on an auxiliary graph Γ(*C*) whose vertex set consists of two copies of the elements of *C*, denoted *v *and v*. Within one copy of *C*, there is an edge between any two elements. The remaining |*C*| edges of Γ(*C*) connect each *v *with its copy v*. The associated edge weights are *ω*_*v',v'' *_=  and *ω*_v,v* _= *S*_*v*_, respectively. An example is shown in Fig. 2.

Clearly, an optimal path of the form *x'*,...,*v'*, *u*, *v''*,...,*x'' *is represented by the edge (*v'*, *v''*) of Γ(*C*), while a self-contained subtree *T*[*v*] is represented by an edge of the form (v, v*). It remains to show that every maximum matching of the auxiliary graph Γ(*C*) corresponds to a legal conformation of paths, i.e., we have to demonstrate that in a maximum matching ℳ, each vertex *v *∈ *C *is contained in an edge. First, note that v* covered by an edge of ℳ if and only if (*v*, v*) ∈ ℳ. Suppose *v *is not covered in ℳ. Since *ω*_v,v* _is non-negative, we can exclude matchings that do not cover all edges of *C *from the solution set. We can thus compute the entries of *S*_*u *_and *Q*_*ux*_, respectively, in polynomial time by solving maximum weighted matching problems with non-negative weights. Introducing the symbol MWM(Γ) for the maximum weight of a matching on the auxiliary graph Γ, we can write this as(7)

Here we make use of the fact that the weight of a matching equals the sum of the weights of the path-systems that correspond to the edges of the auxiliary graphs. In order to facilitate backtracing, we keep tabulated not only the weights but also the corresponding maximum matchings for each Γ(chd(*u*)) and Γ(chd(*u*)\{*u*_*x*_})).

#### Backtracing

Backtracing for multifurcating trees proceeds in analogy to the binary case. Again we start from the root towards the leaves, treating each interior vertex *u*. If |chd(*u*)| = 2, see the backtracing for the binary case. If |chd(*u*)| > 2, we first need the solution ℳ of the MWM for chd(*u*). For each edge (*v*, v*) ∈ ℳ, *v *is called recursively to determine its optimal path-system. Each edge (*v'*, *v''*) ∈ ℳ, however, represents a path *π*_*xy *_that belongs to an optimal path-system. Each of these paths *π*_*xy *_maximizes  for a particular pair of children *v'*, *v'' *∈ *chd*(*u*) and therefore has been stored in *F*_*v'v'' *_during the forward recursion. Thus, each of these paths *π*_*xy *_can be added to the optimal path-system.

As in the binary case, it remains to add the solutions from an optimal path-systems from the subtrees that are not on the path from *x *to *v*' and *y *to *v*″, respectively, for each particular edge (*v'*, *v''*) ∈ ℳ. This can be done as follows. According to eqns.(2) and (4), *R*_*v'x *_can be decomposed into  and either *Q*_*v'x *_or . If |chd(*v'*)| = 2, the child node  that is not on the path from *v' *to *x *is called recursively to obtain an optimal path-system in *T*[*k*]. If |chd(*v'*)| > 2, however, the solution of the MWM for *Q*_*v'x *_is needed to determine an optimal path-system on the subtree , because multiple paths may go through *V*'.  can then be further decomposed until *R*_*xx *_is reached. The same procedure is employed for *R*_*v''y*_.

#### Time and Space complexity

A maximum weighted matching on arbitrary graphs with |*V*| vertices and |*E*| edges can be computed in (|*V*||*E*| log *E*) time and (*E*) space by Gabow's classical algorithm [10] or one of several more recent alternatives [11,12]. In our setting, |*E*| ∈ (|chd(*u*)|^2^), hence the total memory complexity of our dynamic programming algorithm is (*n*^2^).

All entries for  (the edge weights for the matching problems) can be computed in (*n*^3^) time, because a total of (*n *- 1) ∈ (*n*^2^) pairs of leaves have to be considered in eq.(6) and computation of each  entry takes at most (*n*) time. The effort for one of the (|*chd*(*u*)|) maximum weighted matching problems for a given interior vertex *u *with more than two children is bounded by

(|*chd*(*u*)|^3^log(|chd(*u*)|)^2^). The total effort for all MWMs is therefore bounded by

which dominates the overall time complexity of the algorithm (see Appendix for a derivation).

As in the binary case, (*n*^2^) space is necessary and sufficient to store the arrays *R *and *S*. Furthermore, (*n*^2^) space is needed to save the array *Q *and the endpoints (*x*, *y*) of the path *π*_*xy *_that maximized each *Q *entry. The latter is needed for the backtracing. In addition, we keep the quadratic array *n*(*u*, *x*) to allow efficient navigation in *T*. For each interior vertex *u *with |chd(*u*)| > 2, |chd(*u*)| + 1 different maximal matchings have to be stored: one that corresponds to *S*_*u *_and |chd(*u*)| that correspond to *Q*_*ux*_. Each of these solutions requires (|chd(*u*)|) space. The total space complexity of all MWM solutions is therefore ∑_*u*_|chd(*u*)|^2 ^∈ (*n*^2^) (see Appendix).

### Algorithmic variants

Several variants and special cases of the general MPP algorithm are readily derived for related problems. In the following, we briefly touch upon some of them.

#### Special weight functions

It is worth noting that finding a path-system that simply maximizes the number of pairs, as presented in [2] and applied in [13], for example, constitutes a special case of the MPP with unit weights. (Of course the same result is obtained by setting *ω*_*xy *_to any fixed positive weight.) This case may be of practical use under certain circumstances, as it maximizes the number of independent measurements, thus improving power of subsequent statistical tests. Specifically, this weight function selects a path-system with  pairs. In order to maximize the number of edges that are covered by an optimal path-system, we simply set *ω*_*xy *_= *d*(*x*, *y*), where *d*(*x*, *y*) is the graph-theoretic distance, i.e., we interpret the edge lengths in the tree as unity. Alternatively, instead of assigning weights for pairs of leaves directly, edges *e *∈ *E *can be weighted, and the weight for a particular pair of leaves (*x*, *y*) can then be simply defined as .

#### Fixed number of paths

A variant of practical interest is to limit an optimal path-system to *κ *leaf-pairs. This may be relevant in a phylogenetic targeting setting, for example, in cases where resources are limiting data acquisition efforts to a small number taxa so that it pays to make every effort to choose them optimally (see also [4]). Typically, *κ *will be small in this setting.

For binary trees, this variant can be implemented by conditioning the matrices *R *and *S *to a given number of paths. Eq.(2) thus becomes(8)

for a given number *k *≤ *k *in the partial solutions. If an optimal path-system on *T*[*u*] is composed of optimal path-systems on the two trees rooted at its children *v *and *w*, respectively, then the *k *paths are arbitrarily contained within *T *[*v*] and *T *[*w*]. Thus, *k *+ 1 different cases have to be considered, and the case with the highest score has to be identified. This yields to the following extension of eq.(3) for *S*_*u,k*_:(9)

We set *S*_*x *_= *R*_*xx,l *_= *R*_*ux*,0 _= 0 for all *x *∈ *L*, *u *∈ *J*, and *l *∈ {0, *k*}. The latter condition ensures that if no path can be selected anymore in a particular subtree, its score must be 0.

As mentioned above, however, eq.(9) only holds for binary trees. For multifurcating trees, the auxiliary maximum weighted matching problems are replaced by the task of finding matchings that maximize the weight for a fixed number *k *of edges. We are, however, not aware that this variant of matching problems has been studied in detail so far. For small *κ*, it could of course be solved by brute force enumeration.

#### Selecting paths or taxa in addition to already selected paths or taxa

In some applications it may be the case that a subset of taxa or paths is already given, e.g. because the corresponding data have already been acquired in the past. The question then becomes how additional resources should be allocated.

In the simpler case, we are given a partial path-system ∏. It then suffices to remove or mark the corresponding leaves from *T *(to ensure that they are not selected again) and to set the weight of all paths that have edges in common with ∏ to - ∞ to enforce independence from the prescribed pairs.

The situation is less simple if only the taxa are given and the pairs are not prescribed. Here, the goal is to find an optimal path-system that includes all *z *∈ *Z*, where *Z *⊂ *L *denotes the taxa that are required to appear in the output. First, we note that such a solution not necessarily exists, e.g. if |*Z*| = |*L*| and |*L*| is odd. As a simple example, consider a binary tree with three leaves. In that case, only one path and thus two leaves can be selected. This constraint also holds for the subtree rooted at any interior vertex *u *and the *z *∈ *Z *in *T*[*u*], i.e., partial solutions of the MPP (see below).

**Figure 3:**
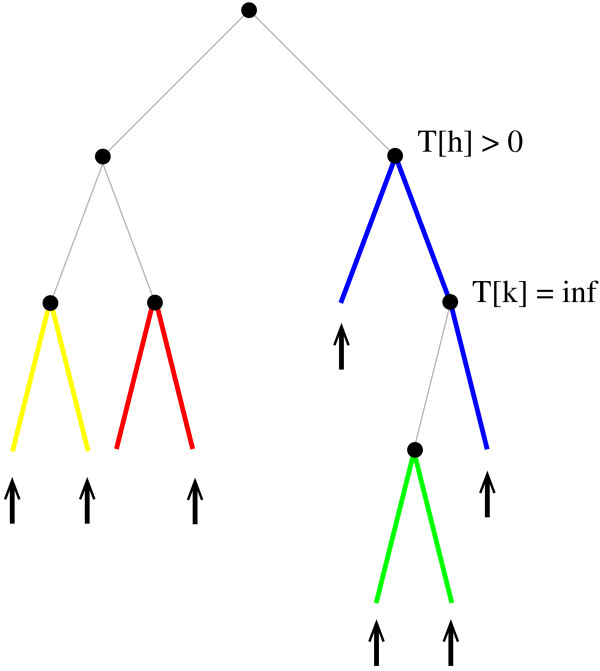
**A binary tree for which only one possible path-system exists that fulfills all constraints**. Leaves that must appear in the output are highlighted with an arrow, and the (only) valid path-system is displayed in color. Note that the score of the subtree *T*[*k*] = ∞, because no path-system in *T*[*k*] exists that includes all three leaves *x *∈ *T*[*k*]. The score of *T*[*h*], however, is greater than 0.

For binary trees, this variant can be implemented by conditioning the matrices *R *and *S *to a subset of all possible paths and leaves. This is achieved by setting the score to -∞ for a particular interior vertex if one of the preconditions cannot be met in eqns.(2) and (3). For example, if two leaves *x*, *y *∈ *Z *have the same father *u*, an optimal path-system of both *T*[*u*] and *T *must contain the path *π*_*xy*_, because otherwise, either *x *or *y *would not belong to the optimal path-system due to the requirement of independence. Similarly, if a particular path *π*_*xy *_in the second alternative achieves the highest score in eq.(3), *π*_*xy *_must not be selected if this conflicts with the possibility to select other prescribed leaves *z *∈ *Z *(Fig. 3).

To derive the recursions for this variant, let *Z*_*u *_denote the leaves *z *∈ *Z *with *z *∈ *T*[*u*] and let *L *be the leaves of *T*[*u*]. It is convenient to first check whether a solution exists for *T*[*u*]. If *L *= *Z*_*u *_and |*L*| is odd, *S*_*u *_= -∞ (i.e., no path-systems exists that selects all *z *∈ *Z*_*u *_in *T*[*u*]). Otherwise, an optimal path-system for *T*[*u*] with *v*, *w *∈ chd(*u*) can be calculated as follows:(10)

Furthermore,(11)

and(12)

for any *x *∈ *L*. In analogy to the algorithm for the unconstrained MPP, we initialize the recursions by *R*_*xx *_= 0 for *x *∈ *L*. This variant does not change the overall time and space complexity, and backtracing is also identical to the unconstrained version of the MPP.

For multifurcating trees, the maximum weighted matching problems are replaced by finding matchings that maximize the weight with the constraint that particular vertices must be included in the matching. Similarly to the variant introduced above, however, we are not aware that this particular problem has been studied in detail.

### Probabilistic version

Sometimes, not only an optimal solution is of interest. As in the case of sequence alignments [14] or biopolymer structures [15], one may analyze the entire ensemble of solutions. Both for physical systems such as RNA, and for alignments with a log-odds based scoring system, one can show that individual configurations ϒ with score *S*(ϒ), in our case path-systems, contribute to the ensemble proportional its Boltzmann weight exp(-*βS*(ϒ)), where the "inverse temperature" *β *defines a natural scale that is implicitly given by the scoring or energy model. In the case of physical systems *β *= 1/*kT *is linked to the ambient temperature *T; *for log-odds scores, *β *= 1; if the scoring scheme is rescaled, as e.g. in the case of the Dayhoff matrix in protein alignments, then *β *is the inverse of this scaling factor. In cases where schemes without a probabilistic interpretation are used, suitable values of *β *have to be determined empirically. The larger *β*, the more an optimal path-system is emphasized in the ensemble. The *partition function *of the system is(13)

The probability *p*_ϒ _to pick ϒ from the ensemble is *p*_ϒ _= exp(-*βS*(ϒ))/*Z*.

The recursion in eq.(3) can be converted into a corresponding recursion for the partition functions *Z*_*u *_of path-systems on subtrees *T *= *T*[*u*], because the decomposition of the score-maximization is unambiguous in the sense that every conformation falls into exactly of the case of recursion. This is a generic feature of dynamic programming algorithms that is explored in some depth in the theory of *Algebraic Dynamic Programming *[16]. We find(14)

with *Z*_*u *_= 1 if *u *∈ *L *and(15)

for *k *∈ *J*. Note that these recursions are completely analogous to the score optimization in eqns.(2) and (3): the max operator is replaced by a sum, and addition of scores is replaced by multiplication of partition functions and Boltzmann factors.

In order to compute the probability *P*_*xy *_of a particular path *π*_*xy *_in the ensemble we have to add up the contributions *p*ϒ of all path-systems that contain *π*_*xy*_(16)

and compute the ratio *P*_*xy *_= *Z*(*π*_*xy*_)/*Z*. The recursions for the restricted partition function *Z*(*π*_*xy*_) can be computed in analogy to eq.(14), but with two additional constraints. First, since *π*_*xy *_∈ ϒ by definition, the leaves *i *∈ *T*[*v*] and *j *∈ *T*[*w*] are constrained in eq.(14), because only paths *π*_*ij *_that are edge-disjoint with *πxy *can be considered. The recursion for the partition function of the last common ancestor node of *x *and *y*, denoted *k*, is also constrained, because *π*_*xy *_must go through *k*. Calculation of the partition functions for the children of *k *is therefore not needed to compute *Z*_*k *_. Thus,(17)

In resource requirements, this backward recursion is comparable to the forward recursion in eq.(3): *Z*(*π*_*xy*_) and thus also *P*_*xy *_can be calculated in (*n*^3^) time, because the number of leaf-pairs that have to be considered is still in (*n*^2^). There is an additional factor (*n*) arising from the need to determine if the path *π*_*xy *_is edge-disjoint with another path, which however does not increase overall time complexity. Furthermore, (*n*^2^) space is needed.

The computation of partition functions is a much more complex problem for trees with multifurcations since it would require us in particular to compute partition functions for the interleaved matching problems. These are not solved by means of dynamic programming; instead, they use a greedy algorithm acting on augmenting paths in the auxiliary graphs. These algorithms therefore do not appear to give rise to efficient partition function versions.

### The TARGETING software

We implemented the polynomial algorithms for the MPP in the program TARGETING. The TARGETING program is written in C and uses Ed Rothberg's implementation [17] of the Gabow algorithm [10] to solve the *Maximum Weight Matching Problem *on general graphs. The software also provides an user-friendly interface and can solve the special weight variants as well. The source code can be obtained under the GNU Public License at http://www.bioinf.uni-leipzig.de/Software/Targeting/.

## Concluding Remarks

In this contribution, we introduced a polynomial algorithm for the *Maximal Pairing Problem *(MPP) as well as some variants. The efficient generalization of the dynamic programming approach to trees with multifurcations is non-trivial, since a straightforward approach yields run-times that are exponential in the maximal degree of the input tree. A polynomial-time algorithm can be constructed by interleaving the dynamic programming steps with the solution of auxiliary maximum weighted matching problems. This generalized algorithm for the MPP is implemented in the software package TARGETING, providing a user-friendly and efficient way to solve the MPP as well as some of its variants.

Future work in this area is likely to focus on developing algorithms for the variants of the MPP on multifurcating trees. In particular, the interleaving of dynamic programming for the MPP and the greedy approach for the auxiliary matching problems does not readily generalize to a partition function algorithm for multifurcating trees. The concept of unique matchings as discussed in [18] may be of relevance in this context.

The MPP solver presented here has applications in a broad variety of research areas. The method of phylogenetically independent comparisons relies on relatively few assumptions [1-3] and is frequently used in evolutionary biology, in particular in anthropology, comparative phylogenetics and, more generally, in studies that test evolutionary hypotheses [19-22]. As highlighted earlier, another application area lies in the design of studies in which tedious and expensive data collection is the limiting factor, so that a careful selection (phylogenetic targeting) becomes an economic necessity [5]. As noted in [13], alternative applications can be found in molecular phylogenetics, for example in the context of estimating relative frequencies of different nucleotide substitutions or the determination of the fraction of invariant sites in a particular gene.

## Appendix

### Pseudocode

Below, we include some pseudocode for the computation of an optimal path-system for an arbitrary tree *T*.

**Require: ***ω*_*xy *_≥ 0 ∀ pairs *x*, *y *∈ *L *and precomputed array *n*(*u*, *x*) *n*(*u*, *x*) ∀ *u *∈ *J *and *x *∈ *L*

1: *S*_*x *_= *R*_*xx *_= *Q*_*x*,*x *_= 0 ∀_*x *_∈ *L*

 if |chd(*u*)| = 2 and  if |chd(*u*)| > 2 ∀_*u *_∈ *J *and *x *∈ *L*

2: **for all ***u *∈ *J *in post-order tree traversal **do**

3:    **if **|chd(*u*)| = 2 **then**

4:      {*v*, *ω*} ← *chd*(*u*)

5:      *S*_*u*1 _= *S*_*v *_+ *S*_*w*_

6:      **for all **paths *π*_*xy *_with *x *∈ *T*[*v*] and *y *∈ *T*[*w*] **do**

7:         determine the path *π*_*xy *_that maximizes

8:         *S*_*u*2 _= *ω*_*xy *_+ *R*_*v,x *_+ *R*_*w,y*_

9:      **end for**

10:      **if ***S*_*u*2 _>*S*_*u*1 _**then**

11:         *F*_*u *_= (*x*, *y*)

12:      **else**

13:         *F*_*u *_= ∅

14:      **end if**

15:      *S*_*u *_= max(*S*_*u*1_, *S*_*u*2_)

16:   **else**

17:      **for all **pairs *v'*, *v'' *∈ chd(*u*) **do**

18:         determine the path *π*_*xy *_that maximizes  and set *F*_*v'v'' *_= (*x*, *y*) and *ω*_*v',v'' *_= 

19:      **end for**

20:      **for all **pairs *v*, v* ∈ chd(*u*) **do**

21:         *ω*_v,v* _= *S*_*v*_

22:      **end for**

23:      use computed edge weights for the following MWM problems

24:      *S*_*u *_= MWM(Γ(chd(*u*)))

25:      **for ***i *= 1 to |chd(*u*)| **do**

26:         *k *← i-th child from *u*

27:         compute *δ *= MWM(Γ(*chd*(*u*)\*k*))

28:         **for all **leaves *x *∈ *T*[*k*] **do**

29:            *Q*_*ux *_= *δ*

30:         **end for**

31:      **end for**

32:      tabulate solution of all MWM problems

33:   **end if**

34: **end for**

The following algorithm summarizes backtracing. It starts at the root of the tree, but consider any vertex *u*:

1: **if **|chd(*u*)| = 0 **then**

2:   return

3: **end if**

4: **if **|chd(*u*)| = 2 **then**

5:   {*v*, *w*} ← *chd*(*u*)

6:   **if ***F*_*u *_= ∅ **then**

7:      call backtracing for *T*[*v*] (using the solution of the MWM for *S*_*v *_if |chd(*v*)| > 2)

8:      repeat for *T*[*w*]

9:   **else**

10:      add *F*_*u *_= (*x*, *y*) = *π*_*xy *_to solution set

11:      *k *= *v *{path from *v *to *x*}

12:      **while ***k *≠ *x ***do**

13:         *

14:         **if **|chd(*k*)| = 2 **then**

15:            call backtracing for 

16:         **else**

17:            call backtracing for *T*[*k*]\*T*[*k*_*x*_] (using the solution of the MWM for *Q*_*kx*_)

18:         **end if**

19:         *

20:         *k *= *k*_*x*_

21:      **end while**

22:      repeat for *k *= *w *{path from *w *to *y*}

23:   **end if**

24: **else**

25:   {*v*_1_, *v*_2_,...,*v*_*n*_} ← *chd*(*u*)

26:   take the appropriate tabulated MWM*M*

27:   **for all **edges (*v*_*i*_, *v*_*j*_)of *M ***do**

28:      add  = (*x*, *y*) = *π*_*xy *_to solution set

29:      *k *= *v*_*i *_{path from *v*_*i *_to *x*}

30:      **while ***k *≠ *x ***do**

31:         see case differentiation for the binary case (lines between *)

32:         *k *= *k*_*x*_

33:      **end while**

34:      repeat for *k *= *v*_*j *_{path from *v*_*j *_to *y*

35:   **end for**

36:      **for all **edges (*v*_*i*_, *v*_l_*)of *M ***do**

37:      call backtracing for *T*[*v*_*i*_] (using the solution of the MWM for  if |chd(*v*_*i*_)| > 2)

38:   **end for**

39: **end if**

### A useful inequality

Consider an algorithm that operates on a rooted tree with *n *leaves requiring ((*d*_*u*_)^α^) time for each interior vertex with *d*_*u *_children. A naive estimate immediately yields the upper bound (*n*^α+1^). Using the following lemma, however, we can obtain a better upper bound. Although Lemma 0.1 is probably known, we could not find a reference and hence include a proof for completeness.

**Lemma 0.1 ***Let T be a phylogenetic tree with n leaves, u an interior vertex*, *d*_*u *_= |chd(*u*)| *the out-degree of u, and α *> 1. *Then*(18)

**Proof **Let *h *denote the total number of interior vertices. Each leaf or interior vertex except the root is a child of exactly one interior vertex. Thus ∑_*u *_*d*_*u *_= *n *+ (*h *- 1). For fixed *h*, we can employ the method of Lagrange multipliers to maximize the objective function  subject to the constraint ∑_*u *_*d*_*u *_= *n *+ (*h *- 1) = *c *≤ 2*n *- 1. The Lagrange function is then(19)

Setting the partial derivatives of Λ = 0 yields the following system of equations:(20)

This system of equations is solved by  for all *i *∈ {1, *h*}. The above sum is maximal when T is a full *d*-ary tree for some *d*. The constraint can thus be expressed as *h *· *d *= *n* + *h - *1 and *F *= *hd*^α ^which is maximized by making *d *as large as possible (i.e., *n*) and hence minimizing the number *h *of interior vertices (i.e., 1). Hence, *F*(*n*)=*n*^α^.

## Competing interests

The authors declare that they have no competing interests.

## Authors' contributions

Both authors designed the study and developed the algorithms. CA implemented the TARGETING software. Both authors collaborated in writing the manuscript. All authors read and approved the final manuscript.
